# Engineered exosomes loaded with miR-449a selectively inhibit the growth of homologous non-small cell lung cancer

**DOI:** 10.1186/s12935-021-02157-7

**Published:** 2021-09-14

**Authors:** Wen Zhou, Mingming Xu, Zhipeng Wang, Mingjun Yang

**Affiliations:** 1 Department of Cardiothoracic Surgery, Affiliated Hospital of Nantong University, No. 20, Xisi Road, Nantong, 226021 Jiangsu China; 2 Department of Thoracic Surgery, Haimen People’s Hospital, No. 253 Renmin West Road, Nantong, Jiangsu China

**Keywords:** Exosomes, miR-449a, Homologous targeting, Non-small cell lung cancer

## Abstract

As an efficient drug carrier, exosome has been widely used in the delivery of genetic drugs, chemotherapeutic drugs, and anti-inflammatory drugs. As a genetic drug carrier, exosomes are beneficial to improve transfection efficiency and weaken side effects at the same time. Here, we use genetic engineering to prepare engineered exosomes (miR-449a Exo) that can actively deliver miR-449a. It was verified that miR-449a Exo had good homology targeting capacity and was specifically taken up by A549 cells. Moreover, miR-449a Exo had high delivery efficiency of miR-449a in vitro and in vivo. We demonstrated that miR-449a Exo effectively inhibited the proliferation of A549 cells and promoted their apoptosis. In addition, miR-449a Exo was found to control the progression of mouse tumors and prolong their survival in vivo. Our research provides new ideas for exosomes to efficiently and actively load gene drugs, and finds promising methods for the treatment of non-small cell lung cancer.

## Introduction

Lung cancer is one of the most common malignant tumors with the highest incidence and fatality rate in the world [1]. According to the Global Cancer Statistics in 2018 [2], there were approximately 18.1 million new cancer cases worldwide, among which more than 2.09 million were the new cases of lung cancer, accounting for 11.6%; in addition, 9.6 million patients died of cancer in 2018, among which patients with lung cancer were approximately 1.76 million, accounting for 18.4%. Lung cancer can be divided into two histological types, including small cell lung cancer (SCLC) and non-small cell lung cancer (NSCLC), among which NSCLC accounts for about 85% of all lung cancer cases. Most NSCLC patients have developed to advanced stages at the time of diagnosis, which brings great challenges to the treatment of NSCLC [3]. In recent years, although targeted therapies, immunotherapy, and the combination of multiple methods have brought new treatment hopes for NSCLC patients, the overall prognosis of NSCLC patients remains poor [4].

Recently, increasing attention has been attached to the relationship between miRNA and NSCLC. MiRNA cleaves mRNA or inhibits translation initiation by imprecisely complementary pairing with the 3′-untranslated region (UTR) of mRNA [5]. It regulates cell differentiation, proliferation, apoptosis, carcinogenesis, hormone secretion and many other biological processes. Many studies have found that there are a variety of miRNA expression disorders in tumor tissues, and these miRNAs may participate in the occurrence and development of tumors by regulating the expression of genes related to cell malignant phenotype [6]. In NSCLC cells or tissues, miR-21 [7], miR-574-5p [8], miR-26 [9], miR-155 [10], miR-1254 [11], miR-449a [12] and other miRNAs affect the progression of NSCLC through cell apoptosis, escaping, cell cycle regulation, infiltration and metastasis regulation, inflammation regulation and other aspects. Therefore, correcting the disordered miRNA in NSCLC cells during the treatment of conventional chemotherapeutics increased the sensitivity of traditional chemotherapeutics and improved the therapeutic effect of NSCLC.

However, miRNA was unstable and easily degraded in vivo, it was a great challenge for choosing efficient and safe gene carriers to effectively transport miRNA into tumor cells and exert curative effect. In recent years, the role of exosomes in cell communication has attracted more and more attention [13–15]. Exosomes are a subgroup of extracellular vesicles with a diameter of 30–150 nm. Exosomes have transmembrane molecules such as CD9 and CD63 on their surface, and contain important genetic materials such as receptors, bioactive lipids, proteins and RNAs [16]. Studies have shown that exosomes transport proteins, functional mRNA, siRNA and miRNA to target cells to perform long-distance cell communication functions, which is an efficient and economical communication mechanism for information exchange between cells [17–20]. Wood et al. [21] have proposed and conceptually verified the feasibility of exosomes as drug carriers for the first time by using immature dendritic cells as parent cells to carry siRNA. Exosome has broad application prospects since it has the advantages of both cell carrier and nano-materials. It can not only ensure the stability of its contents during the transportation process, but also easily penetrate the membrane to achieve intercellular transmission [22, 23]. Nie et al. [24] have prepared a kind of lung-targeted exosomes and use them for the delivery of miRNA-126. They found that the engineered exosome effectively inhibited the proliferation and metastasis of A549 cells, and exhibited anti-cancer effect in nude mouse models. These indicated that exosomes were used as highly efficient vectors carrying specific therapeutic genes for gene therapy of diseases including NSCLC.

There are usually two ways to load miRNA into exosomes [25]. One is to use chemical reagents for transfection to express miRNA in the cell so that the exosomes secreted by the cell will directly package the “cargo”. But this method cannot guarantee that all miRNAs enter into the exosomes instead of adhering to the outer surface of the exosomes. Due to the limited amount of miRNA loaded into the exosomes, the therapy faces inefficiency [26]. Another way is to directly introduce the “cargo” miRNA into exosomes through external forces such as electroporation. Although this method effectively avoids the chemical transfection, some studies have shown that electroporation is easy to cause the aggregation of exosomes and may affect the integrity of exosomes or the carried drugs [27].

Hence, in this research we intended to adopt a method for actively introducing the miRNA “cargo” into exosomes. This method is first proposed by Sutaria et al. [28], using the characteristic of specific binding between transcriptional transactivator protein (TAT) and the trans-activating response (TAR) element to enable the “cargo” miRNA to be efficiently enriched in exosomes. Inspired by this, we constructed an expression plasmid to fuse the TAT peptide with the C-terminus of the membrane protein ADC, and another expression plasmid was constructed to fuse the TAR element with the 5′end of the “cargo” miRNA-449a. After transfection of A549 cells, ADC-TAT and TAR-miR-449a were expressed in A549 cells. During the formation of exosomes in A549 cells, ADC-TAT was inserted into the exosomal membrane and recruited miR-449a that linked to the TAR element through its TAT peptide located on the inner side of the membrane, so that the miR-449a could be efficiently enriched in exosomes. We characterized the collected engineered exosomes secreted by A549 cells, and used A549 cells to verify their high-efficiency uptake ability of self-sourced exosomes. Through a series of experiments, we found that the prepared miR-449a exosomes (miR-449a exo) had strong anti-tumor ability in vitro and in vivo. Our research provided new solutions to the treatment of lung cancer and other cancers with genetic drugs such as miRNA.

## Materials and methods

### Plasmid construction

For the construction of ADC-TAT plasmid, the amplified DNA sequence of ADC was first inserted in pcDNA3.1 expression vector (Miaolingbio, China) between the EcoRI and XhoI sites, and then the TAT peptide synthesized by Sangon Biotech (Shanghai) was inserted to the C-terminus of ADC to obtain pcDNA3.1-ADC-TAT expression plasmid. For the construction of the TAR-miR-449a plasmid, we first obtained the sequence of the miR-449a precursor (pre-miR-449a) from the miRBase database, the primers of pre-miR-449a were designed according to the restriction site (BglII/HindIII) of the pSuper plasmid to be inserted. The sequence of the designed primers was as followed: Forward: 5′-CTAGAGTCAATACAGCTAAC-3′, Reverse: 5′-TCGAACTTGACAGCCATCGA-3′. After PCR amplification, the digested pSuper plasmid (Miaolingbio, China) was ligated with the amplification product that containing pre-miR-449a. The DNA sequence of TAR was synthesized by Sangon Biotech (Shanghai) and then inserted into the 5′end of the pre-miR-449a fragment in pSuper-miR-449a to construct the TAR-miR-449a expression plasmid.

### Cell culture and transfection

The cells used in this research were purchased from the Shanghai Cell Bank of the Chinese Academy of Sciences, and the cells were cultured in RPMI-1640/DMEM medium (Gibco, USA) containing 10% FBS (Gibco, USA) and antibodies (penicillin and streptomycin) (Gibco, USA) and incubated in a 37 ℃, 5% CO_2_ incubator. Cells were collected until the confluency of cells up to 90%, then centrifuged the cells at 1000*g* for 5 min and discarded the supernatant. The cells were finally transferred to the new medium at a ratio of 1:5.

Before cell transfection, the A549 cells in logarithmic growth phase were inoculated in culture plates. When the cells grew to a confluency of more than 95%, pcDNA3.1-ADC-TAT and pSuper-TAR-miR-449a recombinant plasmids (1 µg, respectively) were transfected into A549 cells using the transfection reagent Lipofectamine 3000 (Invitrogen, USA). The transfection reagent and the plasmids were diluted with Opti-MEM medium (Invitrogen, USA).

### Isolation and characterization of the engineered exosomes

Similar methods were applied to isolate and characterize exosomes [29–31]. After cell transfection for 48 h, the supernatant was collected and centrifuged at 4 °C, 500*g* for 5 min to remove the remaining cells. Then centrifugation was performed at 2000*g* for 10 min at 4 °C to remove the residual cell debris from the supernatant. Next, the microvesicles were obtained by centrifugation at 10,000*g* for 30 min. Finally, the supernatant was centrifuged at 10,000*g* for 120 min to obtain exosome precipitation. The supernatant was discarded and the pellet was washed with PBS and centrifuged again to obtain relatively pure exosomes.

After the exosomes were diluted to a lower concentration, the solution was dropped onto the 300 mesh copper net, then the excess liquid was absorbed with filter paper after incubation for 10 min at room temperature. Then, the copper net was stained with 10 µL of 4% uranyl acetate at room temperature for 1 min, dried, and observed the morphology of exosomes by the transmission electron microscope (TEM) (FEI, USA). At the same time, Malvern Dynamic Light Scattering (DLS) analyzer was used to measure the particle size of exosomes.

### Cell uptake of exosomes

In order to evaluate the specific uptake ability of A549 for self-sourced exosomes, we first used the fluorescent dye PKH26 (Umibio, China) to label the extracted engineered exosomes according to the instructions. Then the stained exosomes were co-incubated with A549 cells, Hela cells and HepG2 cells. After 24-h incubation, the nuclei of each group of cells were stained with DAPI. Finally, the laser scanning confocal microscope (Nikon, Japan) was used to observe and record the green fluorescence of PKH26 in each group of cells.

We used flow cytometry to detect the surface marker CD63 of exosomes in each group of cells. In brief, the incubated A549, Hela and HepG2 cells were washed twice with PBS, then added with CD63-FITC monoclonal antibody (Abcam, UK) and incubated at room temperature for 30 min. The fluorescence intensity of CD63 was analyzed by flow cytometry (BD, USA).

### Reverse transcription-quantitative PCR (RT-qPCR)

RT-qPCR was used to evaluate the relative levels of miR-449a in A549 cells and solid tumor tissues after the treatment of exosomes, and to detect the levels of Bcl-2 in cells and tumor tissues to evaluate the inhibition of apoptosis. In brief, we used TRIzol reagent (Invitrogen, USA) to extract total RNA in cells, and then Prime-Script™ RT reagent Kit (Takara, China) was used for RNA reverse transcription, finally the SYBR Premix Ex Taq™ was applied to perform PCR according to the following program: denaturation at 95 °C for 5 min, 95 °C, 10 s; 60 °C, 20 s; 72 °C, 20 s; 78 °C, 20 s (collecting fluorescence), 40 cycles in total. The primers used in PCR amplification were as followed: miR-449a forward: 5′-TACCATTGACAATAGCCGATCG-3′ and reverse: 5′-GACCTAACATTGACTGGAACGATT-3′; U6 snRNA forward: 5′-GACAATCCTAGACTAGCTTACGA-3′ and reverse: 5′-CATGGCACAAGTCATAAGCA-3′. U6 snRNA was used as an internal reference. All primers were synthesized by Sangon Biotech (Shanghai).The relative expression analysis of genes used the 2^−△△Ct^ method.

### Western blot analysis

Firstly, a certain amount of RIPA lysis buffer (Biyuntian, China) was added to the collected exosome pellets or cells or tumor tissues to extract the total protein, and then the total protein of protein concentration was quantitatively analyzed using the BCA kit (Solarbio, China). Subsequently, the loading buffer was added to the sample and boiled for 5 min. After the protein was denatured, it was added to a 12% polyacrylamide gel for electrophoresis separation (constant voltage for 200 V). After that, the separated protein was transferred to PVDF membrane (Millipore, USA) using an electro-membrane transfer instrument. Then, the PVDF membrane was immersed in 5% skimmed milk for 2 h at room temperature, and then incubated with the diluted primary antibody and enzyme-labeled secondary antibody (Abcam, UK) in sequence. Finally, ECL luminescent solution (BBI, China) was added, and the gel imaging analysis system was used to image the protein.

Dilution ratio of the antibodies used in this experiment was as followed: Primary antibodies: anti-CD63 (ab193349), 1:1000; anti-CD9 (ab58989), 1:2000; anti-Bcl-2 (ab117115), 1:5000; anti-Calnexin (ab241154). 1:1000; Secondary antibody: goat anti-mouse (ab182017), 1:2000.

### Cell proliferation detection

The CCK-8 experiment was applied to detect the changes in cell proliferation ability of A549, Hela and HepG2 cells after ingesting the exosomes [32]. Specifically, after being incubated with exosomes, the CCK-8 reagent was added to the cell medium and incubated for 2 h at 37 °C. Then microplate reader (Bioteke, China) was applied to detect the absorbance value of OD_450nm_ at 0 h, 24 h, 48 and 72 h.

In addition, the EdU experiment was carried out to observe the DNA replication in A549 cells more intuitively. The A549 cells incubated with exosomes were stained according to the instruction of EdU Cell Proliferation Kit (Sangon, China), and then the fluorescence intensity in the cells was observed and analyzed with a laser scanning confocal microscope.

### Cell apoptosis detection

Flow cytometry was used to evaluate the apoptosis of A549 cells after ingesting exosomes [33]. Briefly, the incubated A549 cells were first washed with pre-cooled PBS, added with 1 mL binding buffer, centrifuged and removed the supernatant, added 200 µL binding buffer to resuspend the cells. Before stained with PI, cells were filtered once or twice with a 400 mesh screen. Next, we injected 10 µL PI reagents to the cell suspension and mixed them gently, and incubated at 25 ℃ in dark for 30 min. Finally, we calculated the apoptosis rate of each group via flow cytometry.

### Detection of cell migration and invasion ability

Firstly, we used the scratch test to evaluate the changes in migration ability of A549 cells after ingesting exosomes. For this purpose, after the cells were incubated and ingested the exosomes, a sterile pipette was used to make a scratch in each well of the plate and continued to incubate for 48 h. Then we observed and recorded the healing of the scratches in each hole under an optical microscope (Nikon, Japan).

Then transwell experiments were performed to evaluate the migration and invasion capabilities of cells. The A549 cells incubated with exosomes were rinsed and then digested into single cells. They were seeded in a transwell chamber (Corning, USA; 8 μm pore size) without Matrigel on the upper side. After being cultured at 37 °C for 24 h, the cells in the lower chamber were collected and fixed with a fixative for 30 min, and then stained with 0.1% Crystal Violet for 20 min. The migration ability of the cells was evaluated under an optical microscope (Nikon, Japan). The procedure of the transwell invasion experiment was the same as that of the migration experiment, except that the upper side of transwell chamber was precoated with 20 µg/well Matrigel (BD Biosciences, USA; Matrigel: DMEM = 1:4) for 1 h at 37 ℃. Cell numbers were counted in five randomly fields in each Transwell chamber under a light microscope at ×200 magnification.

### In vivo anti-tumor experiment

The Balb/c nude mice were purchased from Laboratory Animal Center of Hangzhou Medical College License No.: SCXK (Zhejiang) 2019-0002, and the animal experiment was approved by the ethics committee of Hangzhou Medical College. A total of 20 Balb/c nude mice were randomly divided into four groups: PBS group, unload Exo group, NC Exo group and miR-449a Exo group. About 1 × 10^7^ A549 cells were injected subcutaneously into the axilla of each mouse for xenograft. After tumor formation, the engineered exosomes were injected through the tail vein of the mouse (2 mg/kg), once every 3 days. Mice were sacrificed via overdose pentobarbital (Sigma, USA; 200 mg per kg body weight (mg/kg); i.p.), and the tumor size of the mice was measured every day for a total of 16 days. In addition, the survival of mice in each group within 40 days was recorded, and the survival period was counted.

### Statistical analysis

RT-qPCR, CCK-8, scratch healing, transwell, flow cytometry and other experiments were repeated for 3 times. All data were analyzed by SPSS 24.0 statistical software. Normally distributed measurement data was expressed as $\bar{\text{x}}$ ± s, the comparison between the two groups adopts *t* test, and the comparison among multiple groups adopts single-factor analysis of variance. *P* < 0.05 or *P* < 0.01 indicated that the difference was statistically significant.

## Results

### Preparation and characterization of engineered exosomes

In order to prepare engineered exosomes that efficiently loaded the gene drug miR-449a, we took advantage of the specific binding between TAT peptide and the TAR element containing a stem-loop structure. In brief, we constructed and expressed two plasmids in the A549 cell. One plasmid contained the membrane localization protein ADC linked to the TAT peptide, and the other contained the miR-449a precursor linked to the TAR element. During the formation of exosomes, ADC-TAT was inserted into the exosomal membrane and recruited miR-449a-TAR through its TAT peptide located on the inner side of the membrane. In this way, miR-449a was efficiently enriched to obtain engineered exosomes.

**Fig. 1:**
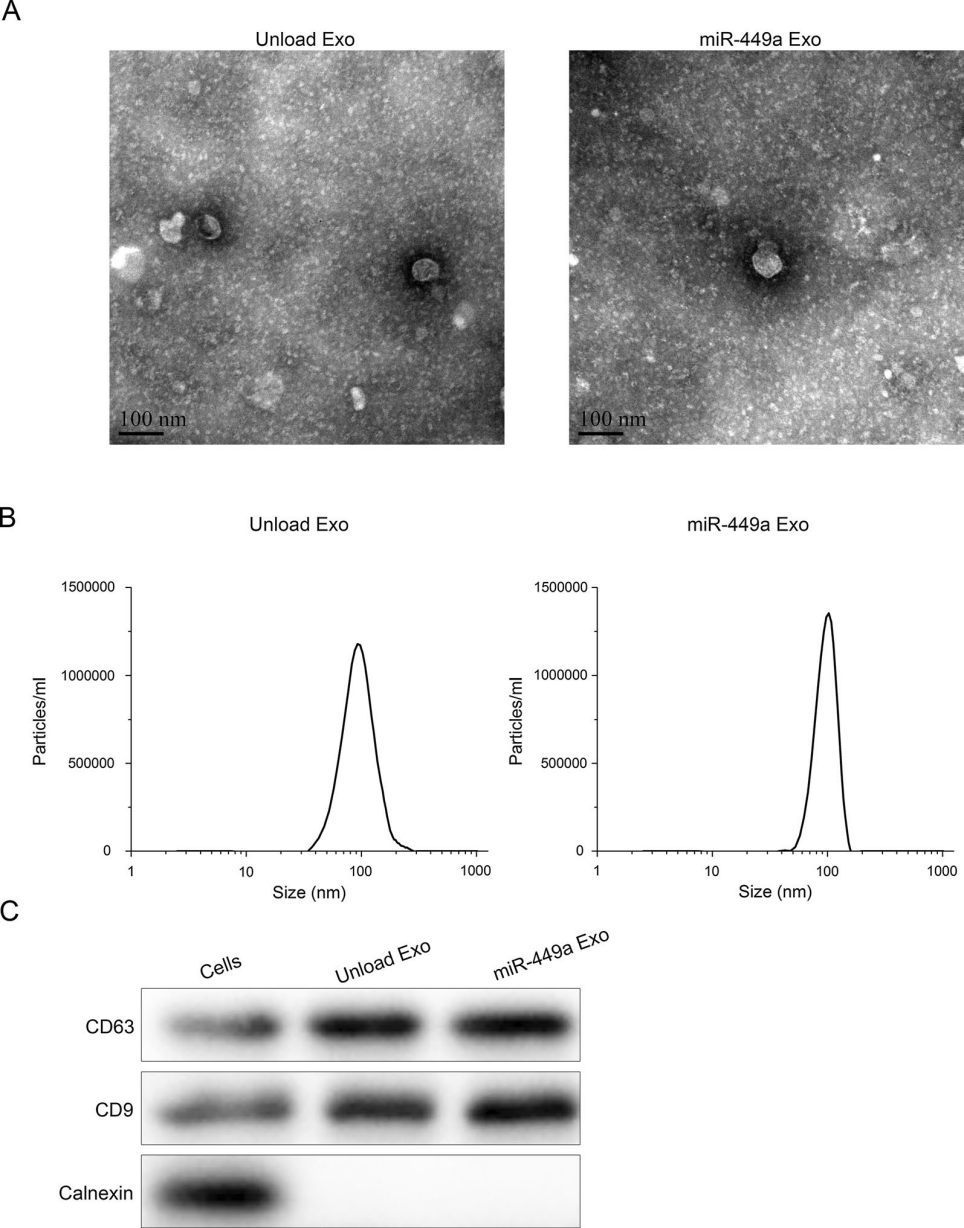
Preparation and characteristic of engineered exosomes. **A** TEM images of the prepared miR-449a Exo and the control Exo. **B** Size distribution of the prepared miR-449a Exo and the control Exo assessed by DLS. **C** Western blot analysis of exosomal surface markers CD63 and CD9

Next, we characterized the prepared engineered exosomes. According to the 2018 proposal of the International Extracellular Vesicle Association (ISEV), exosomes identification methods include transmission electron microscopy (TEM), nanoparticle analyzer (NTA) and protein marker detection. First, we applied TEM and DLS to characterize the morphology and size of exosomes. We observed through TEM that the prepared miR-449a Exo has a spherical vesicle-like structure with a diameter of 90 nm (Fig. 1A). The DLS results showed that the particle size of exosomes was relatively uniform, the average diameter of the unload Exo was 96 nm, while the average diameter of the miR-449a Exo was 104 nm (Fig. 1B). Then, we tested the surface marker proteins CD63 and CD9 of exosomes. The results showed that the surfaces of unload Exo and miR-449a Exo both highly expressed CD63 and CD9, while CD63 and CD9 on the surface of A549 cell membrane showed low expression (Fig. 1C). The above results confirmed that we successfully prepared the engineered miR-449a Exo.

### Specific uptake of self-derived exosomes by A549 cells

**Fig. 2:**
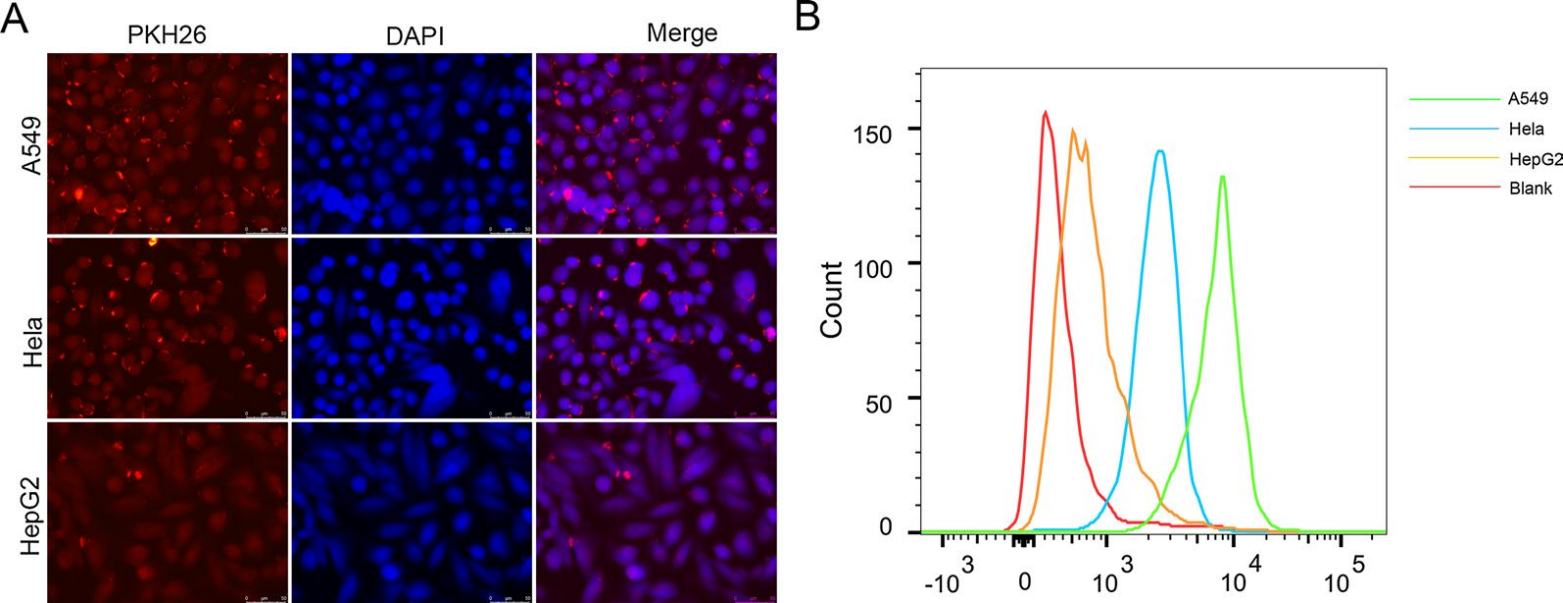
Specific uptake of self-derived exosomes by A549 cells. **A** The uptake of PKH26-labeled miR-449a Exo by A549, Hela and HepG2 cells. Scale bar = 100  nm. **B** Flow cytometric analysis of the content of FITC-CD63, a surface marker of exosomes in A549, Hela and HepG2 cells

We hypothesized that A549 cells had specific uptake capacity for self-derived exosomes. To prove this conjecture, we labeled miR-449a Exo with the fluorescent dye PKH26, and then incubated the PKH26-miR-449a Exo with A549, Hela and HepG2 cells. After ingestion, we used the laser confocal microscope to evaluate the uptake ability of three kinds of cells to miR-449a Exo based on the fluorescence intensity of PKH26. The results showed that the red fluorescence in A549 cells was the strongest (Fig. 2A). At the same time, we conducted flow cytometry to analyze the content of the exosomal surface marker protein CD63 in each group of cells, and indirectly evaluated the uptake ability of miR-449a Exo by different cells. As shown in Fig. 2B, CD63 content was the highest in A549 cells. These results demonstrated that A549 cells had the capacity of specific uptake their self-derived exosomes.

### Effects of miR-449a Exo on the proliferation and apoptosis of tumor cells

**Fig. 3:**
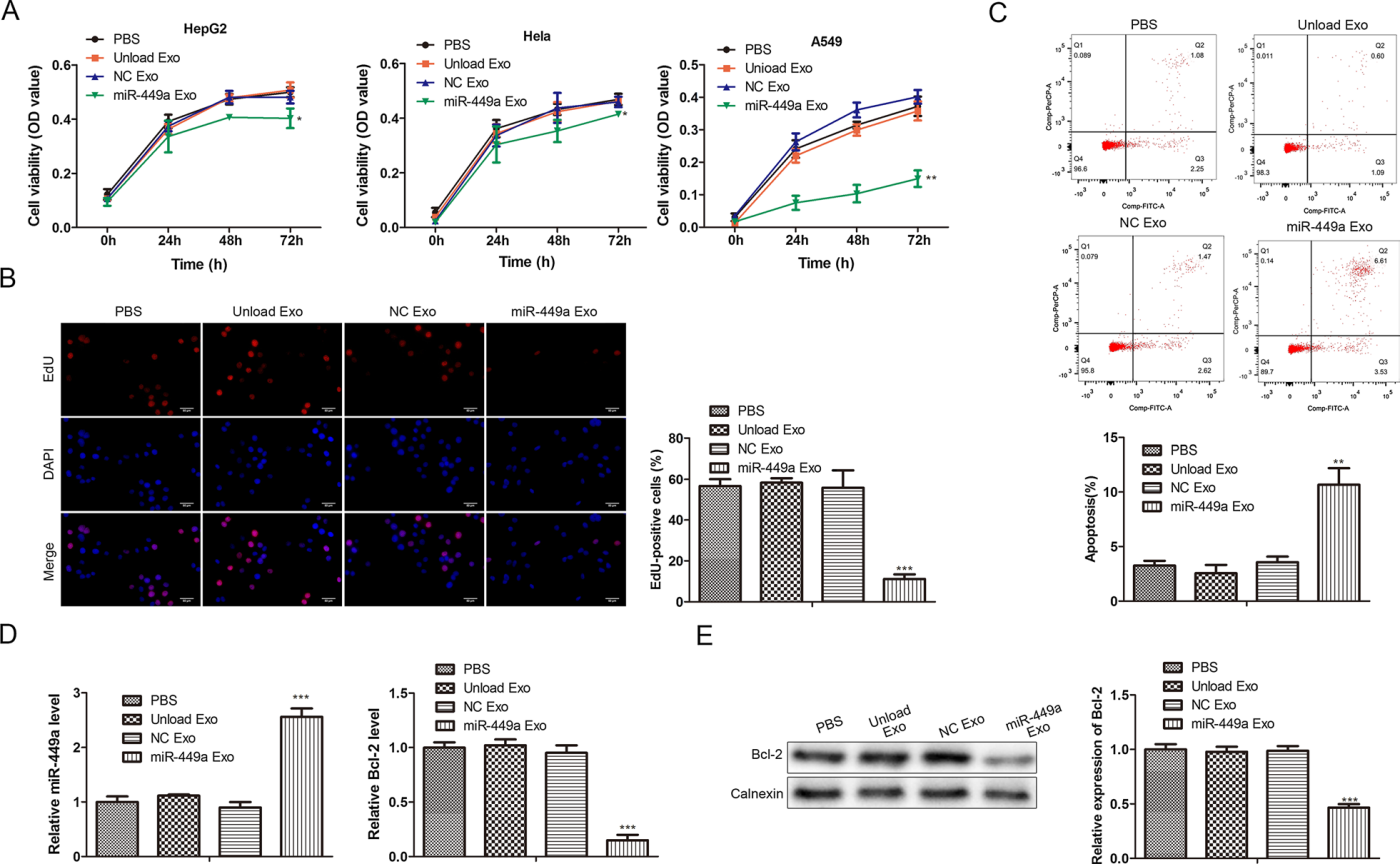
Effects of miR-449a Exo on tumor cell proliferation and apoptosis. **A** The changes in cell viability of A549, Hela and HepG2 cells incubated with miR-449a Exo for 0, 24, 48, and 72 h was evaluated by CCK-8 assay. *P < 0.05, **P < 0.01. **B** EdU staining results of the incubation of A549 cells with exosomes. Scale bar = 100nm. ***P < 0.001. **C** Flow cytometric detection of apoptosis. **P < 0.01. **D** RT-qPCR detection of the relative expression levels of miR-449a and Bcl-2 in A549 cells. ***P < 0.001. **E** The protein expression level of Bcl-2 was evaluated by Western blot. ***P < 0.001

We intended to study whether the prepared miR-449a Exo inhibited tumor development after being taken up by lung cancer cells. To this end, we studied the effect of miR-449a Exo on tumor cell proliferation through CCK-8 and EdU assay. It was seen in Fig. 3A that after the 72-h co-incubation with miR-449a Exo, the cell viability of A549 cells was significantly reduced, while the cell viability of Hela and HepG2 cells did not decrease significantly. In addition, unload Exo and NC Exo had almost no effect on the cell viability of A549 cells, which were the same as the PBS group, indicating that miR-449a was successfully released into the cells and played an anti-tumor effect. The results of EdU staining were the same as above. The proportion of EdU positive cells in the miR-449a Exo-treated group was significantly lower than that of other controls (Fig. 3B). After that, we studied the effect of miR-449a Exo on tumor cell apoptosis. First, flow cytometry was used to detect the apoptosis of the cells in each group. The results showed that the apoptosis ratio of the miR-449a Exo-treated group was significantly higher than that of the other three control groups (Fig. 3C). In addition, we performed RT-qPCR to analyze the relative expression levels of miR-449a and apoptosis inhibitor Bcl-2 in A549 cells. As shown in Fig. 3D, the expression of miR-449a in A549 cells of the miR-449a Exo-treated group was the highest, significantly different from other groups. At the same time, the expression of Bcl-2 in A549 cells of the miR-449a Exo group was significantly lower than that in other groups. Western blot analysis also confirmed the low expression of Bcl-2 in the miR-449a Exo-treated group cells (Fig. 3E). These results indicated that miR-449a promoted the apoptosis of A549 cells.

### Effects of miR-449a Exo on the migration and invasion of tumor cells

**Fig. 4:**
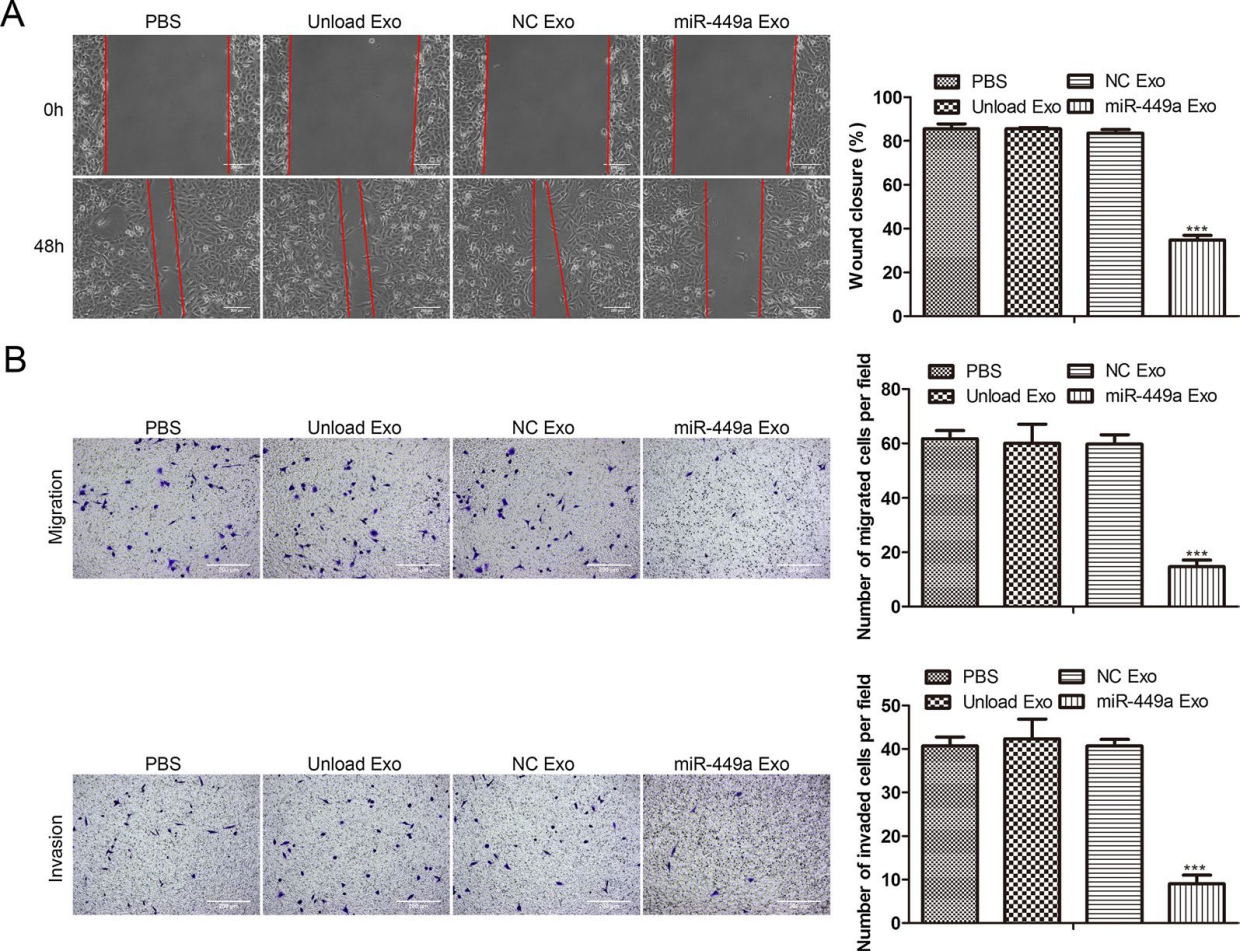
Effects of miR-449a Exo on tumor cell invasion and migration. **A** The influence of miR-449a Exo on the migration ability of A549 cells was assessed via scratch test. ***P < 0.001. **B** Transwell assay was conducted to evaluate the migration and invasion capacity of A549 affected by miR-449a Exo. ***P < 0.001

We further studied the effect of miR-449a Exo on the migration and invasion of lung cancer cells. The scratch experiment results showed that the miR-449a Exo-treated group had the worst scratch healing effect (Fig. 4A), indicating that miR-449a inhibited the migration ability of A549 cells. In addition, we conducted the Transwell experiment, and the results were shown in Fig. 4B. The migration and invasion ability of the miR-449a Exo-treated group was the worst among four groups, indicating that the migration and invasion ability of A549 cells were greatly inhibited by miR-449a Exo treatment.

### Anti-tumor effect of miR-449a Exo in vivo

**Fig. 5:**
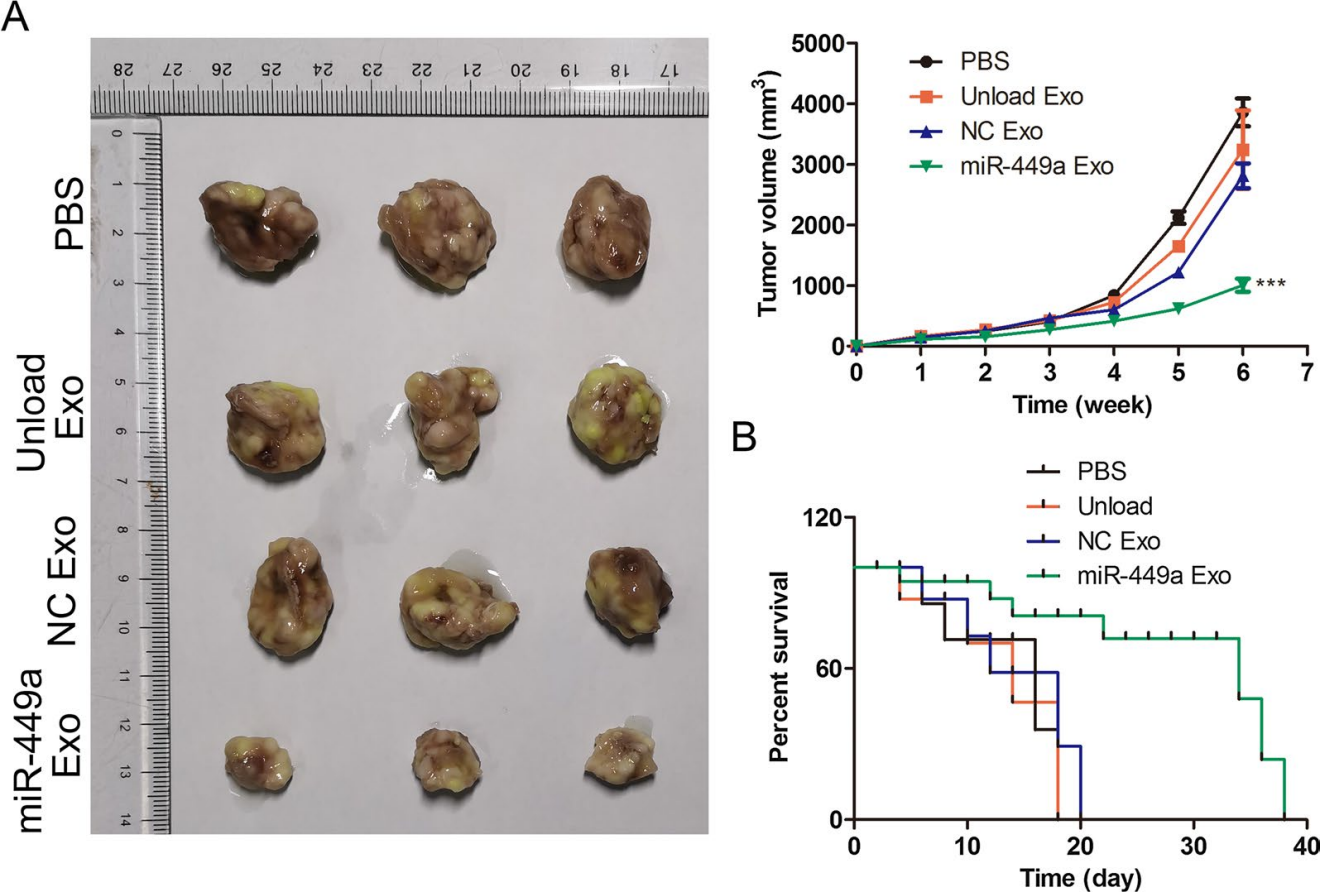
The anti-tumor effect of miR-449a Exo in vivo. **A** Tumor images in different group of mice and the changes in tumor volume of mice treated with different treatments with 16 days. ***P < 0.001. **B** The overall survival of mice in different groups during 40 days

We investigated the anti-tumor effect of miR-449a Exo in vivo by xenograft of A549 cells in a mouse model. By monitoring the tumor volume of mice (Fig. 5A), it was found that the tumor volume of mice in the miR-449a Exo group increased the slowest within 16 days, and there was a significant difference compared with the PBS unload Exo and NC Exo groups (P < 0.05). In addition, by statistical analysis of the survival time of mice in each group, it was found that all mice in the PBS, unload Exo and NC Exo groups died about 20 days after the experiment, while mice in the miR-449a Exo group survived nearly 40 days. The above animal experimental results indicated that miR-449a exhibited anti-tumor effect in vivo and significantly prolonged the survival time of mice.

### Delivery efficiency of miR-449a Exo in vivo

**Fig. 6:**
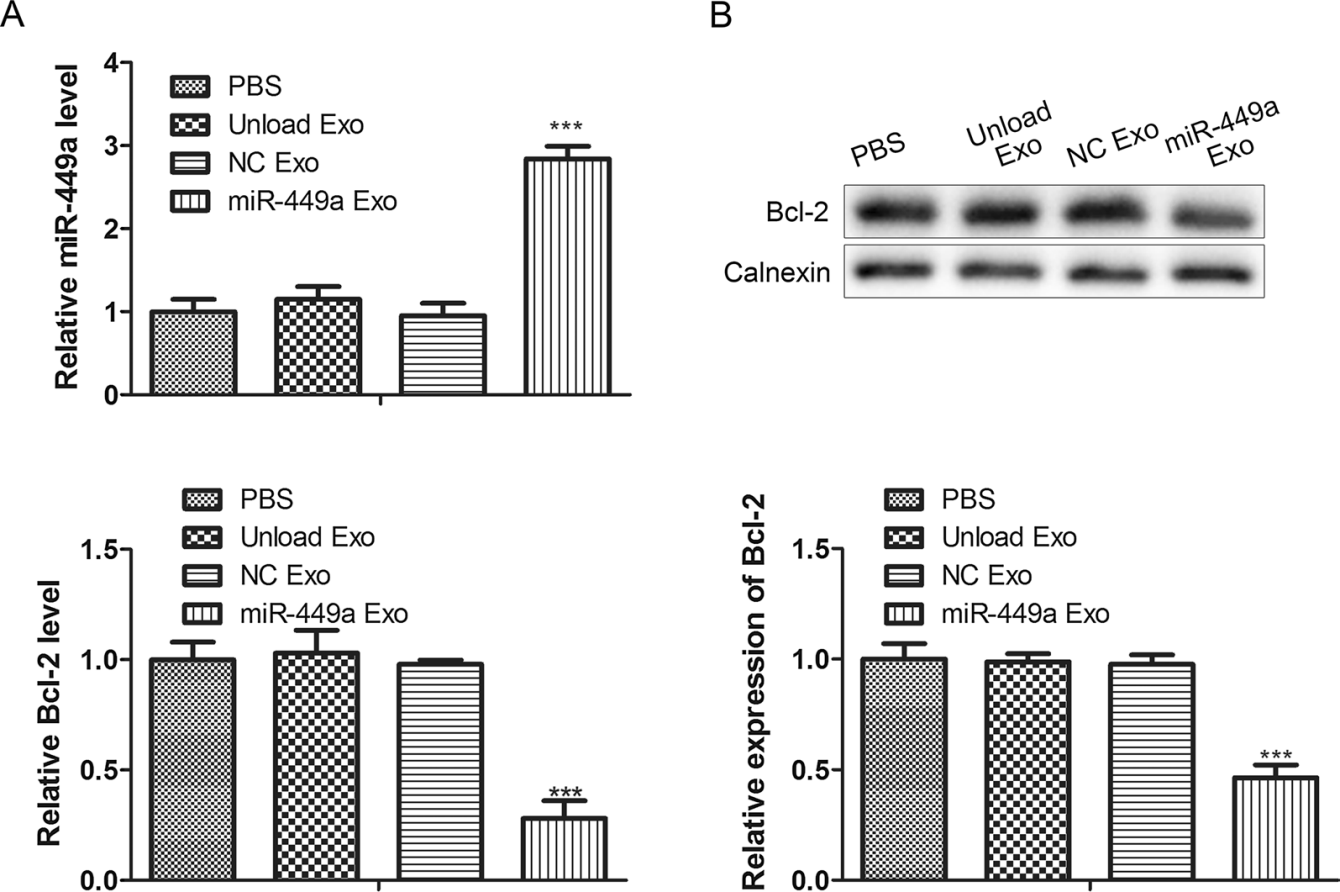
The delivery efficiency of miR-449a Exo in vivo. **A** RT-qPCR detection of the relative expression levels of miR-449a and Bcl-2 in tumor tissues. ***P < 0.001. **B** The protein expression level of Bcl-2 was evaluated by Western blot. ***P < 0.001

In previous experiments, we confirmed that miR-449a Exo had anti-tumor effects in vivo, but we still needed to explore the delivery efficiency of miR-449a Exo in vivo. To this end, we removed and lysed the tumor tissues of each group of mice, and analyzed the expression level of miR-449a in the tumor tissues of the PBS, unload Exo, NC Exo, and miR-449a Exo groups by RT-qPCR. The results showed that the expression of miR-449a in the tumor tissues of mice in the miR-449a Exo group was the highest (Fig. 6A, left), indicating that the delivery efficiency of miR-449a in vivo by miR-449a Exo was higher. In addition, we used RT-qPCR (Fig. 6A, right) and Western blot (Fig. 6B) to analyze the expression level of the apoptosis inhibitor protein Bcl-2 in the tumor tissues of each group of mice. The results showed that the expression of Bcl-2 in tumor tissues of miR-449a Exo group mice was significantly lower than that of the other three groups, *P* < 0.01. These results indicated that the anti-tumor effect of miR-449a Exo in vivo might be achieved by inhibiting the function of Bcl-2.

## Discussion

With the constant development of nanotechnology, nanomaterials had been used in a variety of fields, including the treatment of cancer. As a kind of nano-scale vesicles, exosomes had been widely used as delivery vehicles for various drugs. The specific lipid composition of exosomes provided good stability, thus improving the half-life of the drug in the blood circulation process. At the same time, the lipid composition characteristics of exosomes further stimulated the fusion of exosomes and target cells. In addition, autogenous exosomes reduced the activation of the immune system. Many studies had adopted exosomes to deliver miRNA, siRNA and other gene drugs for the treatment of tumors. Nasari et al. [34] have used exosomes derived from bone marrow mesenchymal stem cells to deliver anti-miR-142-3p to inhibit the expression of miR-142-3p and miR-150 in breast cancer cells, thereby reducing the proliferation ability of breast cancer in vivo and in vitro. Liang et al. [35] have expressed the membrane proteins CD63 and Apo-A1 in 293T cells, so that the collected exosomes own the function of tumor cell targeting. Then, they introduce miR-26a through electroporation to obtain engineered exosomes targeting HepG2 cells, significantly reducing the proliferation and migration rate of HepG2 cells. However, the actual loading efficiency of miRNA loading methods used in these studies is not high, and it is difficult to ensure the integrity of exosomes. In this study, to overcome these problems and actively load miRNA, we used the specific binding between the TAT peptide and the TAR nucleic acid sequence. In brief, through genetic engineering, the membrane protein ADC linked to the TAT peptide and miR-449a linked to the TAR element were expressed in the cells, respectively, so that the exosomes secreted by the cells were “actively” loaded with miR-449a by virtue of the TAT–TAR interaction. MiR-449a is a member of the miR-449 family and has been proven to act as a tumor suppressor by regulating cell proliferation, apoptosis, cell migration and invasion in a variety of cancers [36]. Here, we found that miR-449a inhibited the expression of apoptosis inhibitor protein Bcl-2 in A549 cells, thus promoting cell apoptosis. In addition, the cell proliferation, migration and invasion were promoted by the miR-449a.

As a drug carrier, in addition to effectively loading drugs, targeted delivery is also the key to efficient drug delivery [37]. Many studies have designed specific molecules to be modified on the surface to achieve specific targeting of drug carriers. For example, studies by Hoshino et al. [38] have shown that exosomes derived from tumor cells target different tissues and organs based on the expression of integrins. Among them, the expression of α6β4 and α6β1 on exosomes is related to lung metastasis, and the expression of αvβ5 is related to liver metastasis. Therefore, the expression of integrin on exosomes can be used to predict tumor metastasis. In addition, modification of aptamers or peptides on isolated exosomes can achieve specific targeting of exosomes to cells [39, 40]. In this article, we used homologous tumor cells for the preparation of engineered exosomes to achieve the targeting of exosomes in a more convenient and effective way. Our purpose was to apply exosomes loaded with miR-449a for the treatment of NSCLC, so we used A549 cells for the preparation of engineered exosomes. Homologous targeting experiments proved that the uptake rate of miR-449a Exo prepared by A549 cells was much higher than that of Hela and HepG2 cells. In heterogeneous cancer cells, the targeting capacity of miR-449a Exo was obviously weakened.

In general, we have prepared targeted and engineered exosomes carrying miR-449a, which can be specifically taken up by A549 cells and released miR-449a within the cells to inhibit the proliferation, migration and invasion of lung cancer cells. In addition, we found that miR-449a significantly inhibited the expression of apoptosis inhibitor protein Bcl-2 in A549 cells, thereby promoting cell apoptosis. Animal experiment results showed that miR-449a Exo efficiently delivered miR-449a to mice, which remarkably suppressed the growth of tumor in mice while significantly improved the survival time of xenograft tumor mice. Our research provided new ideas for the application of exosomes in the treatment of lung cancer and other tumors. However, some disadvantages still existed in this research. For example, the engineered miR-449a Exo may have off-target effects in vivo, i.e. attacks on normal organs and tissues, which have not been systematically studied. In addition, there is still a lack of experiments to verify which molecules miR-449a Exo targets to inhibit the proliferation of cancer cells and induce apoptosis. We will gradually improve these problems in the following research.

## Data Availability

All data generated or analysed during this study are included in this published article.
